# Pediatric nutritional surgery and its implications: results from a unicentric retrospective analysis

**DOI:** 10.1007/s00383-024-05700-5

**Published:** 2024-05-02

**Authors:** Girolamo Mattioli, Maria Stella Cipriani, Giulia Barone, Federico Palo, Serena Arrigo, Paolo Gandullia, Stefano Avanzini, Michela Cing Yu Wong

**Affiliations:** 1 Pediatric Surgery Department, IRCCS, Istituto Giannina Gaslini, Largo Gaslini 5, 16147 Genoa, Italy; 2 DINOGMI, University of Genoa, Genoa, Italy; 3 Pediatric Gastroenterology and Endoscopy Department, IRCCS, Istituto Giannina Gaslini, 16147 Genoa, Italy

**Keywords:** Children, Gastroesophageal reflux, Delayed gastric emptying, Dysphagia

## Abstract

**Purpose:**

Existing guidelines provide weak recommendations on the surgical management of nutritional problems in children. The objective was to design a management pathway to address the best nutritional surgery (NS) procedure in a given patient.

**Methods:**

Retrospective analysis of children treated at our department from January 2015 to December 2019. The sample was divided into two groups according to presence or absence of neurological impairment (NI). Patients with NI (Group 1) were classified in three subgroups based on presenting symptoms: A-Dysphagia without gastroesophageal reflux (GER); B-GER with or without dysphagia; C-Symptoms associated with a delayed gastric emptying.

**Results:**

A total of 154 patients were included, 111 with NI. One-hundred-twenty-eight patients underwent only one procedure. Complications and mortality were superior in Group 1. In subgroup A, isolated gastrostomy was the first NS in all patients. In subgroup B most of patients were subjected to a Nissen fundoplication, while in 5 cases total esophagogastric dissociation (TEGD) was the first intervention. Considering the entire sample, 92.3% patients who underwent a TEGD did not require further procedures.

**Conclusion:**

NS encompasses various procedures depending on presenting symptoms and neurological status. A management flowchart for these patients is proposed.

## Introduction

Gastrointestinal (GI) problems are common in children with neurological impairment (NI) and can also affect children with normal neurological development who have a persistent or transient condition causing the issues. GI symptoms that are responsible for nutritional problems and malnutrition include dysphagia, gastroesophageal reflux (GER) symptoms, and symptoms associated with delayed gastric emptying (DGE). Many surgical options are available to cope with these conditions and to provide an adequate caloric intake; we refer to them as Nutritional Surgery (NS) procedures.

This topic is extremely complex to deal with because there are no clear definitions of GI problems that cause nutritional issues, and it is not easy to distinguish them because the symptoms are often similar to each other and overlap. The diagnostic process also varies between different centres. Furthermore, there are no strong recommendations in the literature on when surgery should be indicated and what the best surgical choice is in each case.

In 2017, the European Society for Gastroenterology, Hepatology, and Nutrition (ESPGHAN) published a consensus statement on the management of these issues in children with NI [1]. Then, in 2021 the ESPGHAN conducted a study to determine the impact of these guidelines on clinical practice. Most healthcare professionals caring for children with NI appear to be aware of the recommendations, but considerable variation in clinical practice has been underlined. In conclusion, ESPGHAN highlighted that further studies are required to address open questions and identify knowledge gaps useful for developing updated recommendations and improving patient care [2].

The primary aim of this study was to retrospectively analyse a cohort of patients undergoing different types of NS based on symptom presentation, number and type of surgeries, resolution of symptoms, and outcome in terms of morbidity and mortality, and design a management algorithm to address the best possible surgical option in a given patient.

## Methods

### Design

The study is a unicentric retrospective analysis. The study population includes children (aged 0–18) all treated with NS at our department from January 2015 to December 2019 (5 year period). Having the last NS procedure between 2015 and 2019 at our hospital was inclusion criteria, potentially having one or more NS procedures before 2015.

### Ethical approval

Ethical approval was provided by the Ethical Committee of Gaslini Institute (reference number 003.003.152).

### Setting and patient selection

The analysed population included all patients who were submitted to one or more procedures of NS. NS includes a set of surgical interventions performed with the aim to feed the patient through the enteral way ensuring the best intestinal transit, comprising the procedures aimed at creating direct access to the GI tract for nutritional purposes, those designed to contain the GER and / or to ensure adequate gastric emptying. Procedures done for GI malformations, such as malrotation, hypertrophic pyloric stenosis and intestinal atresia are not included in NS. In our sample, however, we included patients suffering from malformations who underwent an NS procedure after a specific surgery to correct the malformation (e.g., a patient with oesophageal atresia undergoing antireflux surgery after fistula closure and esophageal anastomosis).

The population was divided into 2 groups:

- Group 1: patients with NI, intended as neurodevelopmental delay or impairment;
- Group 2: patients without NI associated with GER symptoms and/or dysphagia.

Patients with NI (Group 1) were furthermore classified as follows:

A. Dysphagia without GER symptoms;
B. GER symptoms with or without dysphagia;
C. Symptoms associated with a DGE with or without other GI symptoms.

Dysphagia was defined by the presence of disturbances in one or more of the three phases of swallowing, typically presenting as feeding difficulties, extended feeding times, malnutrition, and/or a history of aspiration pneumonia [1]. The diagnosis of dysphagia was made by clinical evaluation by a physiatrist and a speech therapist. Some patients underwent video-fluoroscopy.

Typical GER symptoms include chocking, gagging, irritability, regurgitation, refusal to feed, vomiting, and burning pain. Sometimes GER occurs with atypical symptoms including chronic cough, asthma, laryngitis, chronic inflammation of ears and sinuses, bronchitis, and pneumonia [3, 4].

Symptoms associated with a DGE are nausea, vomiting, early satiety, post-prandial fullness and abdominal pain [5].

The types of NS were simplified as follow:

- Gastrostomy alone (gastrostomy performed through open surgery or percutaneous endoscopic gastrostomy);
- Nissen fundoplication (NF) or other antireflux surgery;
- NF associated with gastrostomy;
- Specific surgery for DGE: it includes pyloromyotomy, pyloroplasty, jejunostomy, percutaneous endoscopic transgastric jejunostomy (PEG-J), and gastro-jejunal diversion;
- NF or other antireflux surgery associated with surgeries specific for DGE;
- Total esophagogastric dissociation (TEGD).

A multidisciplinary team, comprising gastroenterologists, physiatrists, anesthetists, radiologists, and surgeons, convenes on a weekly basis to deliberate on patients and potential surgical indications. Subsequently, the team formulates and recommends a comprehensive management program.

## Exclusion criteria:

- Lack of data about symptoms prior to the first surgery;
- Follow-up shorter than 3 months.

## Data collection

Demographic data and disease characteristics were collected for each child, including sex, age at the last surgery, symptoms prior to each surgery, weight-forg-age before the last surgery and at the last available follow-up, presence of NI, isolated skeletal muscular disease, chronic disease, dystonia, epilepsy, inhalation, presence of tracheostomy. Moreover, data on pre- and postoperative diagnostic workup was collected (GI contrast examination, esophagogastroduodenoscopy, gastric scintigraphy, video-fluoroscopy, esophageal pH/multichannel intraluminal impedance monitoring (pH/MII).

Weight-for-age z-score was assessed with the World Health Organization tables for children between 0 and 5 years of age [6], and with the Italian Society of Pediatric Diabetology tables for children between 5 and 18 years of age [7]. 

## Outcome measures

The persistence of the same GI problems or the appearance of new symptoms, the number and type of NS needed for each patient, surgical complications and mortality were evaluated. Complications may necessitate additional surgical interventions; however, these instances are independently evaluated in the analysis.

Morbidity included only major complications (Clavien Dindo grade ≥ 3) [8], divided in short-term complications (< 30 days) and long-term complications (≥ more than 30 days).

Whenever available, twelve-month, 24 month, and/or the last follow-up appointments after the last NS were examined.

Data extraction ended the 31.03.2021.

## Data analysis

Data is presented as frequencies. The Fisher test was used to explore differences between different groups in resolution/or no-resolution of symptoms, complications, and mortality. Significance was defined as *p* ≤ 0.05. Analyses were performed using SPSS^®^ version 26.

## Results

### Entire population

In the 5 year period, 193 patients consecutively underwent one or more NS procedures. Thirty-nine patients were excluded: 8 patients had missing data about preoperative symptoms and 31 had a less than 3 month follow-up. We included 4 patients who had a follow-up of less than 3 months, as they died before 3 months after surgery. The mean duration of follow-up was 29.4 months (median 29.7, range 0.27–72.23).

Patients included in the analyses were 154. Group 1 included 111 patients (72.1%), while group 2 consisted of 43 patients (27.9%).

One-hundred-twenty-eight out of 154 patients underwent only one NS procedure (83.1%), while 26 needed more than one intervention (16.9%). We further explained the types of NS procedures in subgroups. The demographic and clinical characteristics of the patients, the persistence of the same gastrointestinal problems or the appearance of new symptoms at the last follow-up, the short- and long-term surgical complications and mortality for each group and subgroup are shown in Table 1.

**Table 1:** Patients features for each group and comparison of re-intervention rate, persistence or appearance of new gastrointestinal (GI) symptoms at last follow-up, morbidity and mortality between groups and sub-groups. *NI* neurological impairment, *GER* gastroesophageal reflux

	Entire population (*n* = 154)	Group 1 (*n* = 111) patients with NI	Sub-group A (*n* = 46) dysphagia without GER symptoms	Sub-group B (*n* = 51) GER symptoms	Sub-group C (*n* = 14) symptoms associated with a delayed gastric emptying	Group 2 (*n* = 43) patients without NI associated with GER symptoms and/or dysphagia
F	59 (38.3%)	36 (32.4%)	12 (26.1%)	19 (37.3%)	5 (35.7%)	23 (53.5%)
> 1 year*	102 (66.2%)	78 (70.3%)	36 (78.3%)	31 (60.8%)	11 (78.6%)	24 (55.8%)
Epilepsy	59 (38.3%)	58 (52.3%)	27 (58.7%)	20 (39.2%)	11 (78.6%)	1 (2.3%)
Dystonia	45 (29.2%)	45 (40.5%)	22 (47.8%)	18 (35.3%)	5 (35.7%)	0
Inhalation	43 (27.9%)	36 (32.4%)	9 (19.6%)	19 (37.3%)	8 (57.1%)	7 (16.3%)
Tracheostomy	32 (20.8%)	23 (20.7%)	10 (21.7%)	10 (19.6%)	3 (21.4%)	9 (20.9%)
Re-intervention	26 (16.9%)	16 (14.4%)	9 (19.6%)	6 (11.8%)	1 (7.1%)	10 (23.3%)
Persistence/appearance of new GI problems	22 (14.3%)	16 (14.4%)	9 (19.6%)	5 (9.8%)	2 (14.3%)	6 (14%)
Morbidity	17 (11%)	14 (12.6%)	6 (13%)	6 (11.8%)	2 (14.3%)	3 (7%)
Short term	6 (3.9%)	6 (5.4%)	1 (2.2%)	4 (7.8%)	1 (7.1%)	3 (7%)
Long term	12 (7.8%)	9 (8.1%)	5 (10.9%)	3 (5.9%)	1 (7.1%)	0%
Mortality	14 (9.1%)	11 (9.9%)	5 (10.9%)	4 (7.8%)	2 (14.3%)	3 (7%)

* > 1 year of age at the last surgery

*NI* neurological impairment, *GER* gastroesophageal reflux

The prevalence of dysphagia and RGE symptoms in the entire population was 89% and 57.1%, respectively.

Diagnostic investigations were applied in 129 patients before the first NS (83.8%), and in 23 out of 26 patients before the second procedure (88.5%). GI contrast examination was performed on 122 patients (79.2%) before the first surgery and in 23 patients (88.5%) before the second. Before the first NS procedure in 35/122 patients (28.7%) the gastrointestinal contrast examination showed GER and/or DGE.

Twenty-one patients out of 154 underwent videofluoroscopy (13.6%) before the first surgery.

Considering the entire population, seventy-five patients underwent isolated gastrostomy as the first NS procedure, 50 PEG and 25 underwent open gastrostomy. Usually, in our Centre every patient who undergoes PEG has a description of the esophagus and only when there are signs of esophagitis is he subjected to biopsies. In the study population, this information is available in 44/50 patients. They had all described the esophagus and as there were no pathological findings, none of them had performed biopsies.

In our population, body weight and weight percentile before the last NS procedure and at the last follow-up were not available for an individual patient. In 51 out of 154 patients (33.1%) the weight percentile increased at the last follow-up. Twenty-two out of 154 patients (14.3%) showed persistence of the same GI problems (*n* = 9) or the appearance of new ones (*n* = 13) at the last follow-up. Cumulative morbidity after the last surgery was 11% (17 patients), with a short-term complications rate of 3.9% (6 patients) and a long-term complications rate of 7.8% (12 patients). Complications reported were buried bumper syndrome (*n* = 2), slipped NF(*n* = 2), chemical peritonitis due to stomach dehiscence (*n* = 2), adhesive small bowel obstruction (*n* = 2), gastric ulcer, sub-stenosis due to tight NF, stenosis of esophagojejunal anastomosis, peritonitis with necrotising pancreatitis, omental evisceration, esophagojejunal anastomosis dehiscence, splenic injury, volvulus, ascites.

Epilepsy was statistically higher in patients with NI compared to those without NI (52.3% vs 2.3%, *p* < 0.00001). Morbidity was statistically higher in patients with epilepsy (11/59 patients—18.6%) compared to those without epilepsy (*p* = 0.019) (Table 2).

**Table 2:** Comparison of outcome measures between patients with and without epilepsy and patients with and without dystonia

	Entire population (*n* = 154)	Epilepsy (*n* = 59)	Without epilepsy (*n* = 95)	*P*	Dystonia (*n* = 45)	Without dystonia (*n* = 109)	*P*
> 1 year	26 (16.9%)	7 (11.9%)	19 (20%)	0.138	10 (22.2%)	16 (14.7%)	0.183
Persistence or appearance of new GI problems	22 (14.3%)	13 (22.9%)	9 (9.5%)	0.028	7 (15.6%)	15 (13.8%)	0.475
Morbidity	17 (11.0%)	11 (18.3%)	6 (6.3%)	0.019	8 (17.8%)	9 (8.3%)	0.079
Mortality	14 (9.1%)	6 (10.2%)	8 (8.4%)	0.461	3 (6.7%)	11 (10.1%)	0.371

Moreover, 13 patients (22%) with epilepsy had persistence of the same GI problems or the appearance of new ones at the last follow-up (*p* = 0.028). No significant differences were found comparing patients with or without dystonia (Table 2).

A tracheostomy was present in 32 out of 137 patients with dysphagia (23.4%) while this was never encountered in patients without dysphagia (*p* = 0.015).

Fourteen patients died (9.1%), mostly related to comorbidities. The most frequent cause of death was sepsis. Only two patients died within one month of a major surgical complication (jejunal perforation and necrotising pancreatitis) (Table 1).

### Group 1 – Patients with NI (*n* = 111, 72.1%)

Ninety-five out of 111 patients underwent only one NS (85.6%), while 16 needed more than one procedure (14.4%). In detail, 12 patients had two NS procedures, two had 3 and two patients had 4 surgeries, respectively. The specific NS procedures are described in subgroup sections.

### Group 2 – Patients without NI (*n* = 43, 27.9%)

Group 2 included patients with GER symptoms and/or dysphagia without NI; 13 patients (30.2%) with muscular impairment (muscular dystrophies, SMA, myopathies), 7 with oesophageal atresia, 5 with a syndromic condition (Charge, Jeune, Larsen and VACTERL), 4 with chronic disease (cystic fibrosis, Fanconi’s anaemia), 4 with an upper airway disease, 2 with central nervous system tumours, and 8 with other metabolic conditions associated with a nutritional problem.

Thirty-three out of 43 patients underwent a single NS (76.7%), while 10 needed more than one procedure, namely 2 procedures in 6 patients, 3 procedures in 3 patients, and one patient underwent 4 procedures (Table 3). Among patients who needed more than one procedure, the majority had an NF as the first NS procedure.

**Table 3:** Number and type of NS procedures in group 2. The population was divided into 2 groups: group 1 patients with neurological impairment (NI), Group 2 patients without NI associated with GER symptoms and/or dysphagia

	First surgery	Second surgery	Third surgery	Fourth surgery
Isolated gastrostomy	20	3	1	
Isolated Nissen fundoplication	12	6	1	
Nissen fundoplication + gastrostomy	10			
Nissen fundoplication + surgery specific for DGE	1			
Surgery specific for DGE		1	1	1
TEGD			1	
Total	43	10	4	1

*DGE* Delayed Gastric Emptying, *TEGD* total esophagogastric dissociation

### Sub-group A – Dysphagia associated with NI without GER symptoms (*n* = 46, 29.9%)

Thirty-seven out of 46 patients underwent a single NS (80.4%), while 9 patients had more than one procedure. Six patients had two surgeries, one had 3 and 2 patients were operated 4 times.

An isolated gastrostomy was the first surgical procedure in all the patients. Among patients having more than one NS, NF was proposed to 4 patients who were showing persisting symptoms and GER (associated with a gastro-jejunal diversion in one case); TEGD was performed on 4 patients and a PEG-J on the remaining patient. One of these patients had a third intervention (NF re-do) and finally was proposed for a TEGD.

### Sub-group B – GER symptoms associated with NI with or without dysphagia (*n* = 51, 33.1%)

Forty-eight out of 51 patients had dysphagia (94.1%). Forty-five patients of this group underwent a single NS (88.2%), while 6 patients needed more than one procedure. Five patients had two surgeries and one patient underwent three procedures. Most of the patients (*n* = 37, 72.5%) underwent an NF associated with gastrostomy, 6 patients underwent an isolated gastrostomy (11.8%), 2 patients underwent an isolated NF, and one patient underwent an NF plus a pyloromyotomy. Five patients were operated with a TEGD as the first choice.

Six patients had a post-operative persistence of GER symptoms leading to re-intervention. Half of them previously had an isolated gastrostomy as the first procedure, while the remaining 3 previously had an NF plus gastrostomy. Four patients had an NF re-do, one patient a TEGD and one a PEG-J as a second procedure. This latter needed a TEGD as the third NS.

### Sub-group C – Symptoms associated with a DGE with or without other GI symptoms with NI (*n* = 14, 9.1%)

Thirteen out of 14 patients underwent a single NS procedure (92.9%), while only 1 patient needed two surgeries (7.1%).

Ten patients (71.4%) had an NF associated with surgeries specific for DGE (9 patients had pyloroplasty or pyloromyotomy, 1 patient pyloroplasty with gastro-jejunal diversion), 3 patients had an isolated gastrostomy performed (21.4%), and 1 patient a gastro-jejunal diversion. This latter required an NF plus gastrostomy as a second procedure because of GER persistence.

## Discussion

When dealing with GI symptoms, one of the most crucial decisions about the nutritional management of children is whether a non-oral feeding method is suitable to achieve an adequate intake. Often a gastrostomy is indicated not only in cases of dysphagia but also in cases of malnutrition due to other transient or permanent conditions. Most of these patients experience some degree of NI that can significantly worsen symptoms. The management is made even more difficult by the lack of clear indications regarding the diagnostic process, the timing and type of treatment and the management of complications and re-interventions [1, 2].

In the following paragraphs, we present existing knowledge on gastrointestinal issues causing nutritional problems, surgical management, persistence of symptoms, need for reoperation, and associated morbidity. We correlate our findings with established knowledge. Based on our findings and focusing on known information, the multidisciplinary team responsible for the management of these patients in our hospital developed a treatment protocol. This protocol aims to assist in selecting the most suitable surgical option for a specific type of patient, thereby minimizing the need for an excessive number of invasive procedures and reducing potential patient discomfort.

### Symptoms and diagnostic workup

Dysphagia is the most frequent GI symptom complained, with its prevalence ranging from 0.9% in the general pediatric population up to 94% when considering NI children with comorbidities, as in our series [9].

Similarly, typical and atypical GER symptoms are frequently reported by patients and caregivers, leading to GER disease (GERD) in 7–20% of children [10–13]. The prevalence of GER symptoms was 57.1% in our population, obviously it was higher because it represented a sample of patients undergoing NS.

In the absence of warning signs of GER, history and physical examination are usually sufficient [1, 14] However, when symptoms become troublesome or lead to dangerous or long-term complications, ESPGHAN guidelines recommend objective measures such as esophageal pH or pH/MII and/or upper GI endoscopy. In our 5 year experience, 83.8% of patients underwent some diagnostic investigation before the first NS procedure and 88.5% before the second. Most children treated at our Centre were clinically fragile, with complex clinical conditions and multiple comorbidities, therefore if they presented highly suggestive clinical features, symptoms refractory to medical treatment and/or complications of GERD and/or there was another diagnostic test that showed the presence of RGE, after multidisciplinary discussion it may had been indicated to perform reflux surgery even in the absence of a previous GI endoscopy. Considering the entire population, 44 children older than 1 year underwent antireflux surgery isolated or associated with gastrostomy as the first NS procedure, 8/44 patients had had GI endoscopy before surgery (18.2%). Additionally, patients with GERD may exhibit DGE, and the surgical approach may require additional intervention to decrease the risk of fundoplication failure, such as pyloroplasty or gastro-jejunal diversion. Many children had gastrointestinal contrast examination during the study period, we would like to underline that the patients underwent this diagnostic tool to evaluate any anatomical anomalies and not to diagnose GER or DGE.

### Surgical treatment

Multiple studies have shown that patients with nutritional issues often undergo numerous surgical procedures [1, 15–17].ESPGHAN recommends the use of gastrostomy as the preferred method of providing intragastric access for long-term tube feeding in children with NI. The group also advises against performing routine anti-reflux surgery when placing gastrostomy as it may cause significant morbidity [1]. In line with the most recent guidelines, all patients in subgroup A in our study underwent an isolated open gastrostomy or PEG as the first procedure. Guidelines suggest that investigations for GER before PEG placement are not necessary in the case of children without GER symptoms [15]. The correlation between PEG and GERD remains debatable. While some studies concluded that PEG does not exacerbate GERD [16, 17], other authors have proved otherwise [18]. In previous studies, the frequency of additional surgeries for GERD after PEG ranged from 9 to 17% [1, 2, 19, 20],which is similar to the results observed for subgroup A (17.4%).

When a patient presents with GERD Guidelines suggest that anti-reflux surgery should only be considered when other conditions have been ruled out, when symptoms are not resolved by lifestyle changes and medication, and when the patient is at serious risk for a complication [1, 14, 21, 22].Other authors propose that fundoplication to patients with reflux-associated aspiration or moderate to severe esophagitis [2]. In our study, some patients with GER symptoms did not receive anti-reflux surgery as their first operation because they did not meet the above indications. However, some of these patients required a fundoplication as a second procedure, which can be performed around an existing gastrostomy, as demonstrated by Ponsky et al. [19].

The incidence of DGE in pediatric patients with symptomatic GER in the absence of mechanical obstruction is about 50% [23]. Many studies demonstrated that DGE increases the risk of wrap failure after NF in NI children [18]. In subgroup C, pyloroplasty or pyloromyotomy was the most common type of surgery for DGE. Some authors recommend performing pyloroplasty at the same time as fundoplication in patients with DGE. This procedure has been shown to have no effect on morbidity or mortality rates [24]. Gastrojejunal diversion, when combined with fundoplication, may reduce the risk of wrap disruption or herniation by lowering intragastric pressures [25].

Indications for primary TEGD include severe neurodisability (GMFCS-E&R grade V) [26], unsafe swallow, severe GERD unresponsive to medical treatment, recurrent aspiration pneumonia, poor growth, and poor quality of life for both patients and caregivers [27]. Two systematic reviews discuss TEGD in children, with Peters and colleagues [28] collecting 181 cases (157 with NI) and Tanaka et al.[29] including 175 children (147 with NI). Considering both studies TEGD was performed as the primary operation in 60–65% of cases and as a rescue procedure in 35–40%. Recent studies focusing on parents’ perspective report significant improvements in weight gain, reduction in vomiting and regurgitation, airways infections, and hospitalizations for pneumonia. Caregivers also reported feeling more confident and requiring less time in administering food [30, 31]. In our series, patients who underwent either primary or rescue TEGD did not require further NS procedures in 12 out of 13 cases (92.3%).

### Nutritional outcome

Various nutritional data are available in the literature to evaluate malnutrition: feeding time, weight percentile, thickness of the triceps skin fold, medium-upper arm circumference, or muscle area [1]. During data collection for our study, only the weight percentile before and after the last NS procedure was available. In one in three patients in our population the weight percentile increased at the last follow-up.

### Complications

Clavien Dindo grade ≥ 3 complications were collected and analysed. All included patients underwent clinical follow-up at our centre. There may be potential bias in the reporting of postoperative complications. Apparently, no patients underwent surgery for complications in local hospitals.

The complication rates associated with gastrostomy fashioning have been reported to range from 16 to 70%[32] depending on the placement technique [33]. In our study, 75 patients underwent an isolated open gastrostomy or PEG as their first NS, with a morbidity rate of 9.3%.

NF most common complication is dysphagia, [21, 22, 34, 35] other common complications are gas-bloat, early satiety/pain, retching, dumping syndrome, affecting nearly 50% of patients.[36]Other complications are worsening aspiration risk from oesophageal stasis, and wrap slipping/unwrapping [1]. In our population, 14 patients underwent an NF alone as the first surgery while 73 patients to an NF associated with other procedure, and the morbidity after these procedures was 14.3% and 11%, respectively.

Early complication rate for TEGD is 16%, with 7–8% of cases requiring surgical intervention, while late complications occur in 15–19% of cases. The re-operation rate is comparable to and even lower than re-operation rates following fundoplication. The overall mortality rate associated with complications of TEGD is reported to be between 1.5 and 3.3% [27–29].However, it is important to underline that in our series as in the literature, the mortality rate reflects the natural progression of the underlying conditions.

### Persistence of symptoms and need for re-operation

After their last NS procedure, 14.3% of patients in our study reported the persistence of the same GI symptoms or the development of new ones. In larger published studies, recurrence rates following fundoplication range from 10 to 25% in NI children, compared to 2% to 10% in children with normal neurological development [18, 37].

As previously described, children with NI affected by seizures represent significant risk factors for redo Nissen [38].

In our sample, epilepsy was significantly more frequent in the NI group and the presence of epilepsy was correlated with a greater incidence of morbidity and persistence/appearance of new gastrointestinal problems. Studies have reported failure rates ranging from 6.8 to 52% for redo NF [25, 36, 39–42].

In our patient series, only 16.9% of patients required more than one NS procedure. Comparing the two groups, we observed a higher re-intervention rate in Group 2 (19.6% vs 14.4%, *p* = 0.142), although this result may be influenced by the fact that this group is smaller. The literature typically reports re-intervention rates for specific types of NS.

While NF after PEG is reported in 9–17% of patients in the literature [1, 2, 19, 20], there are no published reports on the rate of other types of NS following isolated PEG/gastrostomy procedures.

In a comparison of NF and PEG-J, children who underwent PEG-J had a higher incidence rate of redo interventions than those who underwent fundoplication [43]. The possibility of establishing post-pyloric nutrition (PEG-J or jejunostomy) could overcome the problem of DGE, but the incidence of complications from these procedures is not negligible [44].

## Conclusion

**Fig. 1:**
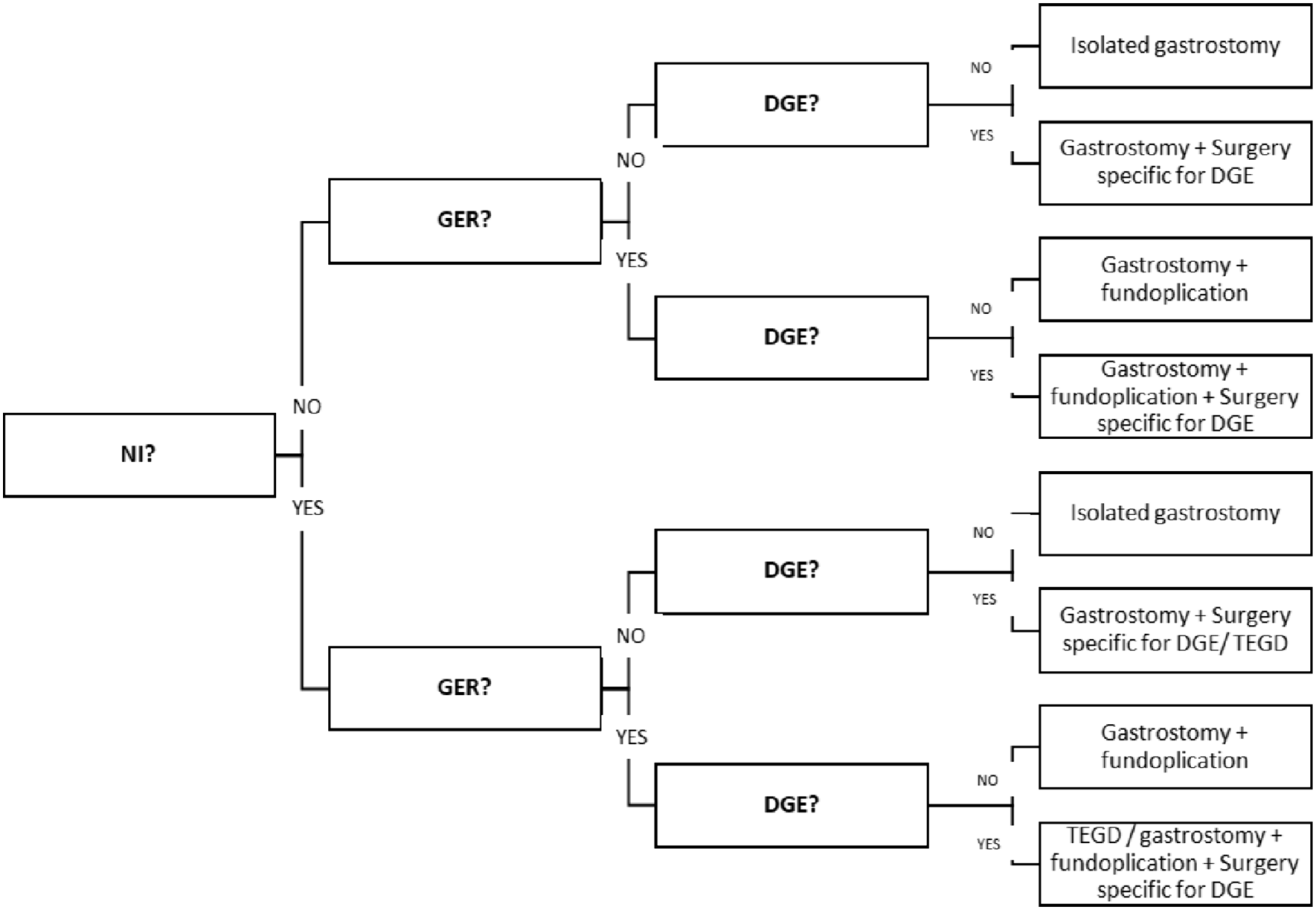
Nutritional Surgery flowchart for patients with dysphagia

On the basis of this experience, the multidisciplinary team drawn some therapeutic indications in the management of patients who require nutritional support. Faced with the presence of dysphagia defined by the need to dedicate more than 3 h a day to nutritional management, it is appropriate to ask whether and which conditions exist, including NI, the presence or absence of GER and/or DGE. Each combination of symptoms may determine the choice of an NS procedure and any salvage procedures in case of persistence of symptoms or treatment failure. (Fig. 1) We are currently using this flowchart to guide therapeutic choices when an indication for NS is given.

This paper exhibits several strengths. Firstly, it addresses the need for studies on the management of nutritional problems in children, a gap identified by ESPGHAN, highlighting the importance of additional research to answer unresolved questions and identify knowledge gaps for updated recommendations. Additionally, the paper introduces, for the first time, a definition of Nutritional Surgery. The retrospective analysis includes all patients undergoing NS procedures over a five-year period, providing a valuable foundation for formulating hypotheses in future research. Notably, the study examines a diverse range of NS procedures in a large patient cohort, respecting the heterogeneity of the population undergoing such procedures. Furthermore, the inclusion of patients without NI as a distinct group is a noteworthy departure from existing guidelines. The paper also presents a management protocol developed by a multidisciplinary board based on both the study’s results and the existing literature. However, certain weaknesses should be acknowledged, including the lack of initial nutritional status data for patients who underwent procedures in other centres, making comparisons challenging. The heterogeneity in group sizes and patient characteristics further complicates group and sub-group comparisons. Lastly, as a retrospective analysis, the study can only present a therapeutic pathway followed for these patients, rather than designing a definitive therapeutic algorithm.

## Suggestions for future research

Since we cannot design a therapeutic algorithm based on a retrospective study, we would suggest for future research a prospective multicentre study that systematically analyse the clinical and nutritional status of patients, and that uses the scheme we designed as the study protocol for assigning patients to a specific NS procedure. Once the patients have been assigned, it will be possible to evaluate the outcome and see if the rate of NS re-operation is reduced compared to the data currently reported in the literature.

The culmination of such research efforts could lead to the formulation of robust recommendations and the development of a diagnostic-therapeutic algorithm, providing valuable guidance for clinical professionals in managing cases of NS.

## Data Availability

No datasets were generated or analysed during the current study.

## Abbreviations

DGE: Delayed gastric emptying
ESPGHAN: European society for pediatric gastroenterology hepatology and nutrition
GER: Gastro-esophageal reflux
GERD: Gastro-esophageal reflux disease
GI: Gastro intestinal
GMFCS: Gross motor function classification system
NF: Nissen fundoplication
NI: Neurological impairment
NS: Nutritional surgery
PEG: Percutaneous endoscopic gastrostomy
SMA: Spinal muscular atrophy
SPSS: Statistical package for the social sciences
TEGD: Total esophagogastric dissociation
